# Phytonutrients: Sources, bioavailability, interaction with gut microbiota, and their impacts on human health

**DOI:** 10.3389/fnut.2022.960309

**Published:** 2022-08-16

**Authors:** Juntao Kan, Feng Wu, Feijie Wang, Jianheng Zheng, Junrui Cheng, Yuan Li, Yuexin Yang, Jun Du

**Affiliations:** ^1^Nutrilite Health Institute, Shanghai, China; ^2^Sequanta Technologies Co., Ltd., Shanghai, China; ^3^Department of Molecular and Structural Biochemistry, North Carolina State University, Kannapolis, NC, United States; ^4^Chinese Center for Disease Control and Prevention, National Institute for Nutrition and Health, Beijing, China

**Keywords:** phytonutrients, gut microbiota, metabolism, health outcomes, bioavailability

## Abstract

Phytonutrients are natural bioactive components present in the daily diet that can exert a positive impact on human health. Studies have shown that phytonutrients may act as antioxidants and improve metabolism after being ingested, which help to regulate physiological processes and prevent metabolic disorders and diseases. However, their efficacy is limited by their low bioavailability. The gut microbiota is symbiotic with humans and its abundance and profile are related to most diseases. Interestingly, studies have shown that the gut microbiota is associated with the metabolism of phytonutrients by converting them into small molecules that can be absorbed by the body, thereby enhancing their bioavailability. Furthermore, phytonutrients can modulate the composition of the gut microbiota, and therefore improve the host's health. Here, we focus on uncovering the mechanisms by which phytonutrients and gut microbiota play roles in health, and the interrelationships between phytonutrients and gut microbiota were summarized. We also reviewed the studies that reported the efficacy of phytonutrients in human health and the future directions.

## Introduction

Nutrients play an essential role in maintaining the regular functions in the body. Their basic functions include providing energy, contributing to body structure, and regulating chemical processes. Carbohydrates, lipids, protein, vitamins, and minerals are the major nutrients in food (1, 2). There are also certain physiologically active compounds known as “phytonutrients” that play a crucial role in human health. Previous studies have shown that phytonutrients are effective in preventing and mitigating a variety of diseases and physiological disorders, thus imposing a tremendous impact on the medical and health care system. Common phytonutrients include polyphenols, phytosterols, saponins, and carotenoids. Well-documented phytonutrients, such as catechins, curcumins, anthocyanins, quercetin and chlorogenic acid, can be easily ingested from diets, and many studies have demonstrated their role in anti-cancer, neuroprotection, anti-aging, treatment of metabolic disorders, and other diseases (3–5). For example, curcumin and chlorogenic acid have been implicated in alleviating fat accumulation, high cholesterol, and metabolic disorders by affecting protein synthesis pathways and modulating immune responses (6, 7). In general, due to people's pursuit of a healthy diet, there has been a growing focus on natural products in recent years (8), and phytonutrients hold a great promise in healthcare and clinical therapy thanks to their beneficial effects (9, 10).

Understanding the metabolism and pharmacokinetics of phytonutrients may facilitate the application of phyto-pharmaceuticals to mitigate various diseases. Limited by phytonutrients and individual differences in digestive capacity, membrane transporters and metabolic enzymes, only a small part of phytonutrients can be directly absorbed by the human body after oral administration, and metabolism is mainly carried out in the liver and intestine (11). In the liver, phytonutrients undergo oxidation, demethylation, and hydrolysis reactions in phase I, and then combine with endogenous binders in phase II to form small molecules to facilitate absorption. Despite a low inaccessibility in the upper gastrointestinal tract, the bioavailability of phytonutrients can be greatly enhanced with the participation of gut microbiota in the lower gastrointestinal tract (12). Poorly absorbed phytochemicals undergo microbiota-mediated biotransformation such as cleavage, demethylation, dihydroxylation, deglycosylation, and are able to produce metabolites with higher bioavailability and bioactivity (13, 14). For example, the bioavailability of cyanidin-3-glucoside is only 0.02%, while the microbial degradation product of cyanidin-3-glucoside, 3,4-dihydroxybenzoic acid, has a bioavailability of 44% (15).

Gut microbiota is a complex ecological community comprising trillions of bacteria that live in the human gut and develop a mutually beneficial symbiosis (16). Gut microbes assist in human digestion by decomposing chemicals that the human gut is unable to degrade on its own (17). *Firmicutes, Bacteroidetes, Actinobacteria*, and *Proteobacteria* are the most common bacteria genera in the human gut microbiota (18). They can integrate brain and gastrointestinal functions, such as intestinal movement, appetite and weight, through the microbiota-gut-brain axis (19), and then affect normal physiology and disease susceptibility through their collective metabolic activity and host interactions (20–23). Gut microbiota are not static, instead, they have been in a dynamic process throughout the lifespan (24).

The relationship between phytonutrients and the human gut microbiota is a two-way complex interaction. Phytonutrients are absorbed first to alter the composition of the gut microbiota which includes inhibiting pathogens and promoting the growth of beneficial bacteria (5), and then to influence the production of their metabolites, which would further modify the intestinal environment by inhibiting the production of harmful compounds such as indole, lipopolysaccharide, and hydrogen sulfide (25). Some polyphenols, such as those found in green and black tea, may inhibit the growth of detrimental bacteria such as *Helicobacter pylori, Listeria monocytogenes, Staphylococcus aureus, Escherichia coli, Salmonella typhimurium*, and *Pseudomonas aeruginosa* (26–29). In the meantime, microbial metabolites such as short chain fatty acids and other bioactive components fermented/degraded by gut microbiota not only provide essential materials and energy for the growth of gut microbes, but also can target multiple pathways in intestine, liver, and pancreas, resulting in improvements in gut health (8, 30). For example, chlorogenic acid and its related compounds can be metabolized in the gut by the resident microbiota, which are responsible for the release of caffeic acid and further metabolism, producing phenyl–propionic, phenylacetic, and benzoic acid derivatives that are then absorbed into the circulatory system for further actions (7, 31).

Overall, the relationship between phytonutrients and human health is established through the metabolic function of the human digestive system, mainly through the participation of gut microbiota (32, 33). The gut acts as an important sensory organ capable of detecting, transmitting, integrating, and responding to signals from the internal and external environment, thereby triggering responses. Cascades of communication along the gut-brain axis are associated with inflammatory responses and immune homeostasis (34). For example, Ohno et al. reported that curcumin supplementation modulated the composition of gram-negative bacilli and subsequently strengthened intestinal barrier and regulated the metabolic functions of gut microbes, such as bile acid biosynthesis and arachidonic acid metabolism (35). The purpose of regulating physiological processes and treating diseases is achieved through immunomodulation and anti-inflammatory, anti-oxidative stress, and inhibition of various enzymatic functions (6, 36), showing a complete chain of action of phytonutrients. Accumulating evidence shows that different phytonutrients may impact human health through different modes of action and targets (37–42).

**Figure 1:**
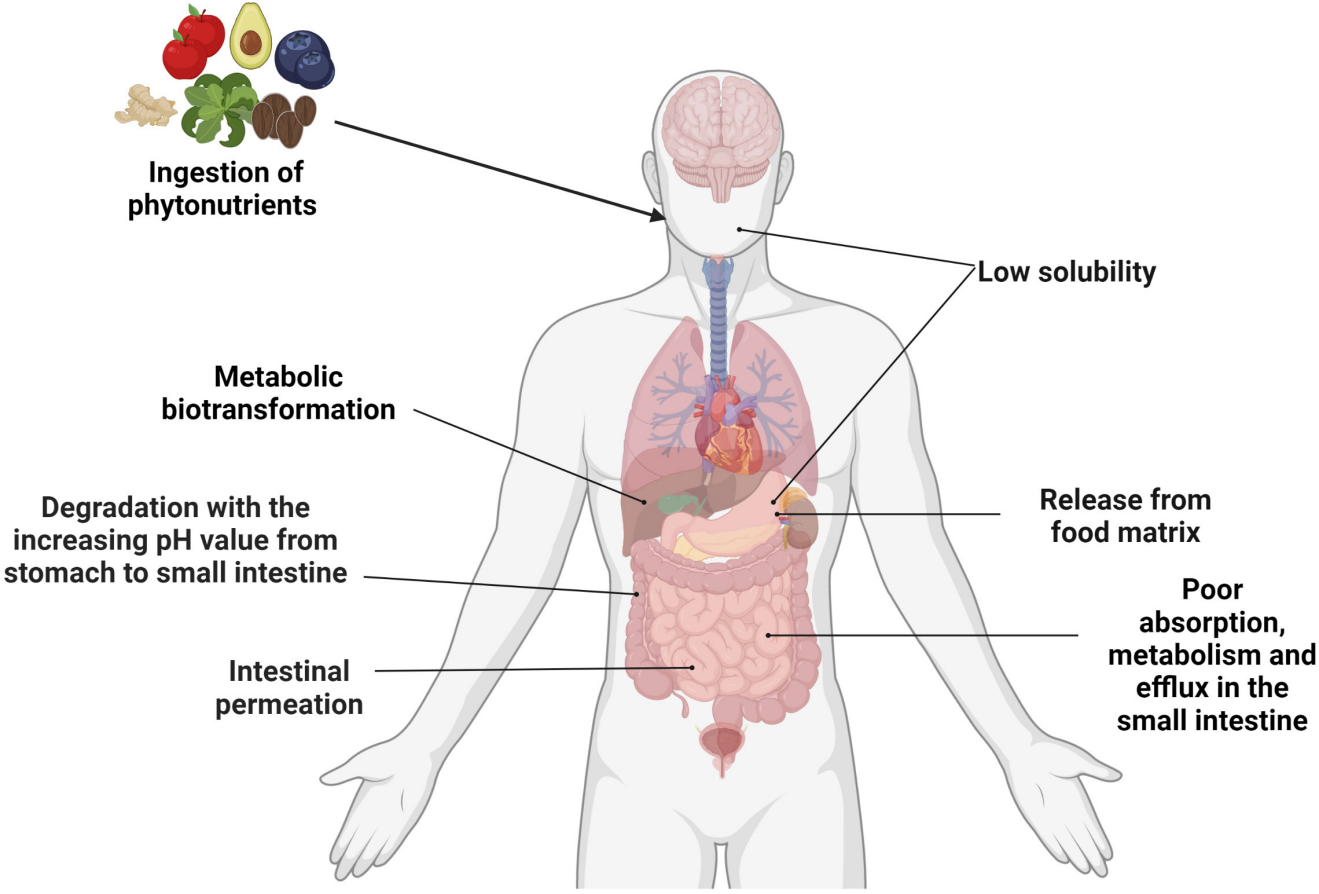
Summary of the factors affecting the bioavailability of phytonutrients. Created with BioRender.com.

In conclusion, as the natural products from plants, phytonutrients have shown unique diversity and safety. Phytonutrients are involved in various physiological processes and may prevent/mitigate disease pathogenesis through the gut microbiota, which are closely linked with an individual's overall health (Figure 1). The complex and dynamic interactions between phytonutrients and gut microbiota have become a research hotspot. This review summarizes the sources, metabolic processes of five phytonutrients including catechins, curcumin, quercetin, anthocyanins and chlorogenic acid, and their interactions with the gut microbiota. In addition, the influence of these impacts on metabolism and the future directions of phytonutrient-related studies are portrayed in detail.

## Catechins and gut microbiota

### Sources and chemical structures of catechins

**Figure 2:**
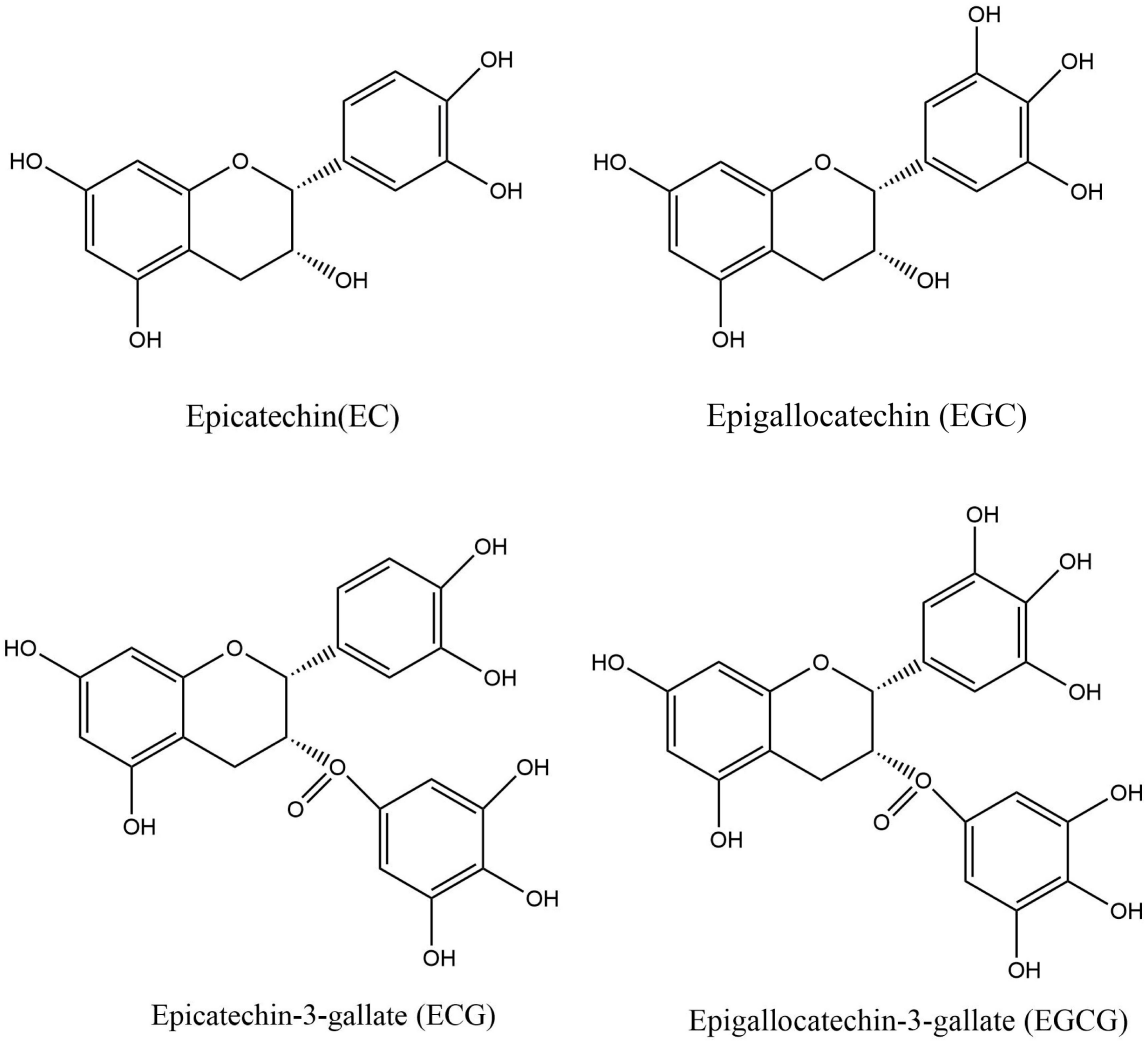
Structure of EC, ECG, EGC, and EGCG.

As a member of the flavan-3-ol polyphenol family, catechins are widely distributed in a range of dietary sources, such as tea, cocoa, apple, and kiwi fruit (43, 44). Catechins are extremely abundant in polyphenol-rich green tea and its extracts, accounting for approximately one-third of the solids in brewed green tea (45, 46). Catechins include four major items, namely epigallocatechin-3-gallate (EGCG), epigallocatechin (EGC), epicatechin (EC), and epicatechin-3-gallate (ECG) (Figure 2), among which EGCG is the most abundant and has the highest biological activity (47, 48). The presence of certain chemical structures like catechol group and pyrogalol group in these compounds provide them with strong antioxidant properties and biological activity (49). Numerous studies have demonstrated the health effects of catechins, including anti-inflammatory, antimicrobial, immunomodulatory, and neuroprotective effects (50).

### Bioavailability and metabolism of catechins

The bioavailability of catechins in the human body is extremely poor (51). After oral administration, only a small fraction of catechins is bioavailable (52), and transported to the liver *via* the portal vein, where phase II enzymes convert them into methyl, glucuronide, and sulfate derivatives. In fact, only around 14% EGC, 32% EC, and 0.1% EGCG are accessible upon oral intake (53).

Approximately two-thirds of catechins reach the colon where catechins are degraded by microbial enzymes into a range of metabolites (54, 55), followed by being released into the enterohepatic or systemic circulation to perform diverse physiological roles (56). There is evidence indicating that the phase II metabolism of EC may occur in enterocytes, and the metabolites (mainly sulfated conjugates) of EC were eliminated by efflux back to the intestinal lumen, which was much higher than the elimination *via* bile (57).

### Interactions between catechins and gut microbiota

Biotransformation of catechins into their metabolites is mainly dependent on the gut microbiota. The gut microbiota's vast gene pool transforms the colon into a bioreactor with immense catechin metabolic capacity (58, 59).

**Figure 3:**
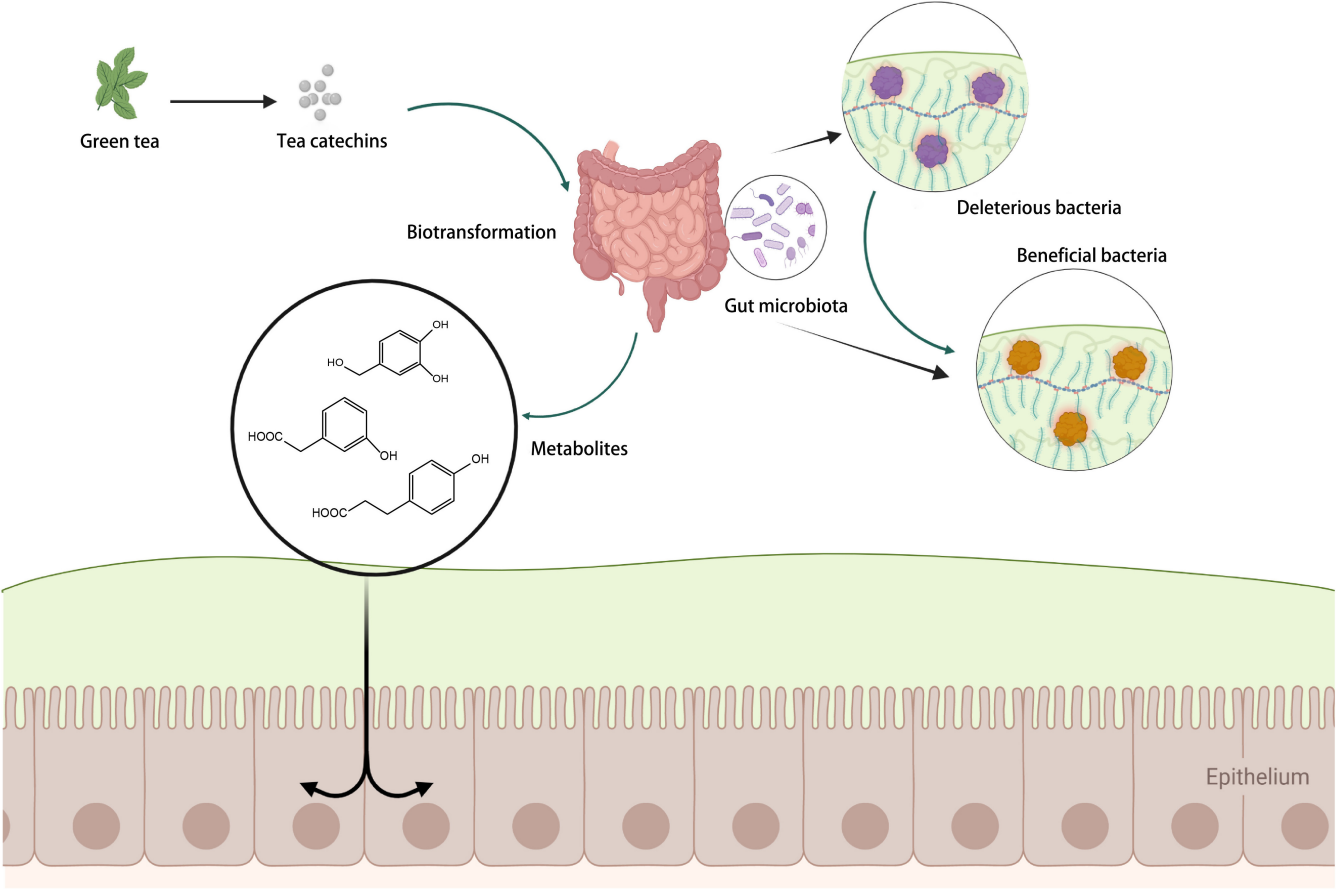
The regulation of catechins on gut microbiota. Created with BioRender.com.

The gut microbiota can execute glycosidic connections, C-ring fission, and degradation of the heterocyclic structures of catechins, resulting in smaller compounds such as phenylvalerolactones and phenylvaleric acids (55). These newly produced microbial metabolites may then be absorbed across the colon epithelium and eventually enter the systemic blood circulation (41). The metabolites of catechins produced by microbial biotransformation may even outperform the parent molecules in terms of biological activity (60). The relevant process is summarized in Figure 3.

### Metabolism of catechins by gut microbiota

Catechins are metabolized by the gut microbiota to facilitate absorption, and the degradation pathway has been documented in various studies. The transformations can be split into three major processes: (I) galloyl ester hydrolysis, (II) C-ring opening, and (III) lactonization, decarboxylation, dehydroxylation, and oxidation processes to further modify the reaction products (53, 61, 62). Regarding galloylated catechins (ECG and EGCG), the microbial metabolism begins with galloyl ester hydrolysis by microbial esterases, which produces gallic acid. The C-ring of the catechin residue is opened after degalloylation, resulting in diphenylpropan-2-ol, which is then transformed into valerolactone, resulting in the formation of hydroxyphenylvaleric acids and/or 4-hydroxyphenylvaleric acids. Meanwhile, dehydroxylation of these hydroxylated phenolic acids at the original B-rings may occur, yielding a variety of simpler phenolic acids. The principal reaction products from catechins for intestinal absorption are valerolactone and phenolic acids (63), which can be easily absorbed by the large intestine and then undergo phase I and phase II metabolism, distribution and excretion (53).

Kutschera et al. reported that two bacterial strains, *Flavonifractor plautii* and *Eggerthella lenta*, could biotransform dietary catechins into hydroxyvaleric acid and valerolactone metabolites (64). To further understand the underlying mechanisms of the gut microbiota in modulating the effects of catechins on health, more research is required to determine the microbiota's functional guild and characterization (65).

### Modulation of gut microbiota by catechins

Catechins are antibacterial compounds that are effective against foodborne and other harmful microorganisms. The most widely acknowledged mechanism for catechins' antibacterial action is their capacity to disrupt membranes. EGCG can attach directly to the exposed peptidoglycan layer of Gram-positive bacteria, causing cross-links in the peptidoglycan to be cleaved. When the peptidoglycan layer is damaged, its protective impact is diminished and permeability increases, resulting in bacterial demise (53, 66).

Several *in vitro* studies suggest that catechins may have prebiotic effects by selectively improving the growth of beneficial gut microbiota (28, 67). Green tea catechins have been shown to boost the populations of beneficial bacteria, such as *Bifidobacterium* spp. and *Lactobacillus*, while suppress the harmful bacteria including *Clostridium* spp. in the *in vitro* fermentation tests. Catechins have also been found to increase the variety of gut microbiota and reduce the ratio of *Firmicutes*/*Bacteroidetes* in several animal studies (68, 69). Liao et al. found that tea catechins significantly increased the abundance of *Bifidobacteria* while lowering serum total cholesterol and low-density lipoprotein cholesterol levels in mice (70). Furthermore, dietary catechins have been reported to increase the relative abundance of *Akkermansia Muciniphila* and alleviate high fat diet-induced metabolic syndrome (71).

### Catechins, microbiome composition, and related health benefits

Obesity is the outcome of energy surplus and is a major risk factor for a variety of chronic diseases. As a result, implementing a nutritional intervention to avoid obesity has become a public health priority (72, 73). Studies have depicted that obese individuals have a lower gut community diversity and different microbial profile compared with their lean counterparts (74). The involvement of tea catechins in weight management has been proposed due to their beneficial effects in modulating gut microbial compositions. Hursel et al. reported that catechins may improve blood biomarkers for metabolic syndrome, such as insulin, glucose, low-density lipoprotein, which was accompanied with an altered *Firmicutes* to *Bacteriodetes* ratio (67). In rats, the combination of catechins with a high fructose oligosaccharide (FOS) diet reduced their body weight, which was correlated with increased *Parabacteroides* spp., *Phascolarctobacterium* spp., *Robinsoniella* spp., *Prevotella* spp., and decreased *Lachnospira* spp., *Clostridiales* spp., *Ruminococcus* spp., *Peptococcaceae* spp., and *Oscillospira* spp. (75).

A recent study suggests that catechins may have prebiotic-like activity and therapeutic potential through modulating gut microbiota. Notably, the abundance of *Bacteroides* and *Firmicutes* in the intestinal mucosa of patients with inflammatory bowel disease (IBD) was significantly reduced after catechin administration, while the population of *Proteobacteria* and *Actinobacteria* was significantly increased (76). Therefore, understanding the underlying crosstalk mechanism may help us to further elucidate the clinical value of tea catechins in the prevention and treatment of IBDs (77).

Patients with non-alcoholic fatty liver disease (NAFLD) exhibit lower *Bacteroidetes* and *Firmicutes* abundance and lower gut microbiota diversity than healthy people. The severity of non-alcoholic steatohepatitis has been linked to changes in microbiota composition (78). Patients with cirrhosis and hepatocellular carcinoma, for example, showed higher levels of *Bacteroidetes* and *Ruminococci* and lower levels of *Bifidobacterium* than those with cirrhosis alone. The link between gut dysbiosis and NAFLD, as well as the fact that catechins lowered endotoxin levels in the circulating and portal circulation, highlights the preventative and therapeutic potential of catechins in restoring gut barrier integrity and reducing the hepatic and intestinal inflammation (46).

## Curcumin and gut microbiota

### Sources and chemical structures of curcumins

Curcumin, also known as diferuloylmethane (1,7-bis(4-hydroxy-3-methoxyphenyl)-1,6-heptadiene-3,5-dione), is a polyphenol representing the major curcuminoids extracted from the rhizomes of Zingiberaceae and Araceae plants. It is the main active ingredient of turmeric, a common Asian spice used as a dietary spice, food coloring, and herbal medicine (47, 79). Different biological and pharmacological aspects have sparked widespread interest in its therapeutic potential. Its bioactive constituents have recently been studied. It was reported that curcumin, has showed potent functions at the cellular level *via* regulating numerous signaling pathways (80). Notably, an increasing number of clinical trials with regard to curcumin supplementation have been published or are presently underway, reflecting the scientific community's growing interest in curcumin's therapeutic potential (79, 81–84).

### Bioavailability and metabolism of curcumins

Because of the poor solubility, low bioavailability, chemical instability, and rapid metabolism, curcumin's therapeutic potential is severely limited (85, 86). Curcumin undergoes substantial metabolism (reduction, sulfation, and glucuronidation) in liver, kidney, and intestinal mucosa after oral administration (87). Liver is the major site where the metabolism of curcumin occurs, whereas intestine and gut bacteria also play an important role in facilitating this process. In hepatocytes and enterocytes, the double bonds of curcumin are reduced, resulting in dihydrocurcumin, tetrahydrocurcumin, hexahydrocurcumin, and octahydrocurcumin (88, 89). The intestinal microflora is capable of deconjugating phase II metabolites and converting them back to phase I metabolites leading to the production of fission products (e.g., ferulic acid) that have a higher bioavailability (90).

In spite of the low digestibility in the upper gastrointestinal tract, curcumin can be fermented by gut microbiota, which potentially explains how it performs various physiological activities.

### Interactions between curcumins and gut microbiota

**Figure 4:**
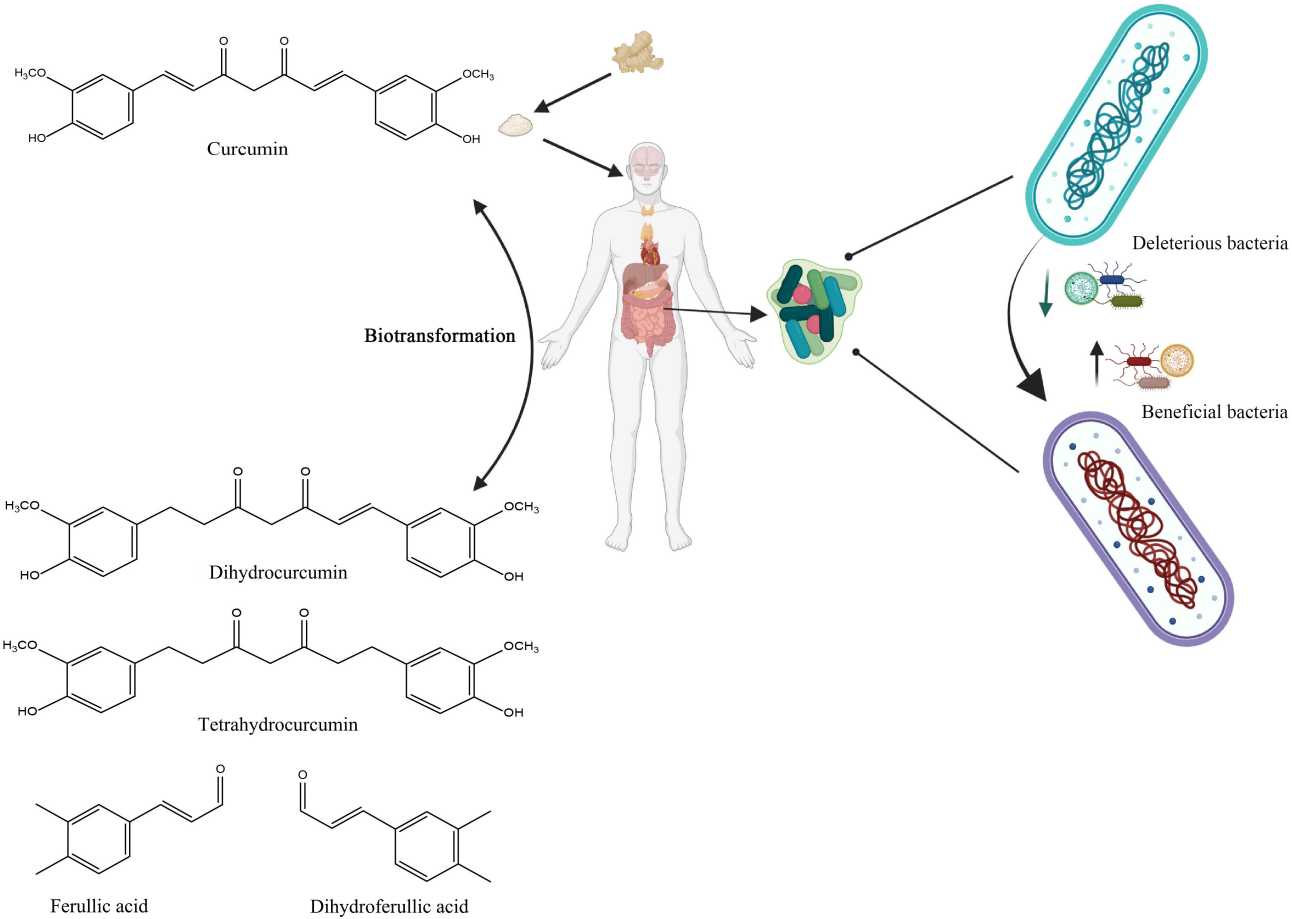
Schematic illustration of the bidirectional interactions between curcumin and gut microbiota. Created with BioRender.com.

Curcumin bioactivity, like that of other dietary polyphenols, is linked not only to absorption rate but also to its gut microbial digestion. Curcumin has shown preferential distribution and accumulation in the gut after oral or intraperitoneal dosing, despite its limited plasma and tissue bioavailability (79, 91). Curcumin can be transformed by human intestinal microbiota in a variety of metabolic pathways, including the production of active metabolites with local and systemic effects, but also by reducing the heptadienone backbone and demethylation by *Blautia* spp (92, 93). In summary, undigested curcumin may accumulate in the gut, where upon the fermentation by microbiota, it can be converted into biologically active metabolites, which subsequently modulate the growth of gut microbiota in a selective manner (Figure 4).

### Metabolism of curcumins by gut microbiota

Because of its formidable metabolic functions, such as the transformation of numerous compounds that reach the colon, the gut microbiota can be described as a biological reactor. Since the biotransformaiton of phytonutrients is achieved with the microbial enzymes produced by the gut microbiota, the efficiency of curcumin biotransformation differs by microbial profile (84, 94).

Curcumin can be modified in the colon tract by a specific microorganism, *Escherichia coli*. Curcumin/dihydrocurcumin reductase (CurA) is a nicotinamide adenine dinucleotide phosphate (NADPH)-dependent enzyme that converts curcumin to the intermediate product dihydrocurcumin and then to the final product tetrahydrocurcumin. By reductive disruption of the chromophoric diarylheptatrienone chain in the first process of the reaction, dihydrocurcumin is produced from curcumin. The mechanism for converting dihydrocurcumin to tetrahydrocurcumin is the same in the second step (95).

Multiple gut bacteria are involved in curcumin metabolism, among which the *Firmicutes Blautia* spp. produces two derivatives, demethylcurcumin and bisdesmethylcurcumin, through demethylation (92). While *Escherichia fergusonii* and *Escherichia coli* strains are involved in the metabolic processes that produce dihydrocurcumin, tetrahydrocurcumin, and ferulic acid (82). Other microorganisms capable of degrading curcumin include *Bifidobacterium longum, Bifidobacterium pseudostrandum, Enterococcus faecalis, Lactobacillus acidophilus*, and *Lactobacillus casei*. Microbial metabolism of curcumin by *Pichia anomala* or a bacterial strain of *Bacillus megaterium* has been reported to produce new metabolites *via* various metabolic processes such as hydroxylation, demethylation, reduction, and demethoxylation. An ultra-performance liquid chromatography analysis identified 23 metabolites and discovered several novel human gut microbiota-curcumin metabolic pathways (96).

Interestingly, curcumin metabolites have been shown to have an equivalent or stronger potency compared with the parent compound. Tetrahydrocurcumin, for example, outperforms curcumin as a free-radical quencher which has shown therapeutic effects in alleviating neurodegenerative diseases (97).

### Modulation of gut microbiota by curcumins

Since a majority of curcumin escapes the upper gastrointestinal digestion and reach the colon, it may exert pharmacological effects by altering the richness, diversity, and composition of gut microbiota (98). A previous study found that curcumin-treated patients had a 69% increase in bacterial species, whereas an overall 15% decrease in bacterial species was observed in the control group. Participants who responded to the treatment had consistent increases in *Bacteroides* spp., *Clostridium* spp., *Citrobacter* spp., *Enterobacter* spp., *Enterococcus* spp., *Klebsiella* spp., and *Pseudomonas* spp., as well as decreased relative abundance in several *Blautia* spp. Curcumin increased the abundance of *Bifidobacteria, Lactobacilli*, and butyrate-producing bacteria while decreasing *Prevotellaceae, Coriobacterales, Enterobacteria*, and *Enterococci*, resulting in a significant shift in the beneficial-pathogenic microbiota ratio. Aside from anti-inflammatory and anti-colonotropic carcinogenicity activities, changes in gut microbiota may partially explain how curcumin regulates immune responses and mitigate hyperlipidemia (93).

By using a mouse model of acute myeloid, Liu et al. revealed significant changes in gut microbiota composition by intravenous curcumin administration. Specifically, they observed a significant increase in *Lactobacillus acidophilus, Bifidobacterium bifidum* and *Lactobacillus reuteri*, and decrease in pathological bacteria including *Bacteroides fragilis, Escherichia coli, Fusobacterium nucleatum* and *Akkermansia* in the interventional group, which was associated with a reduced disease severity (99). In rats, curcumin lowered the *Firmicutes/Bacteroidetes* ratio and reversed the high fat diet-induced gut dysbiosis, which was related to a decreased hepatic ectopic fat deposition, reduced inflammatory markers, and enhanced intestinal barrier integrity, suggesting that it could be a novel therapeutic strategy for NAFLD (100).

### Curcumin, microbiome composition, and related health benefits

Changes in the microbiome have been linked to a variety of metabolic diseases, including obesity, diabetes, and chronic liver disease (101). In a rat model of menopause, Zhang et al. investigated the relationship between curcumin intake and gut microbial diversity, and reported that curcumin might partially repair the changes in the richness and composition of the rat gut microbiota caused by ovariectomy-induced estrogen shortage (102). In specific, curcumin increased the abundance of *Serratia, Shewanella, Pseudomonas, Papillibacter*, and Exiguobacterium species, while lowered that of *Anaerotruncus* and *Helicobacter pylori* (80).

Curcumin has shown neuroprotective properties by targeting the gut-brain axis and reducing intestinal inflammation through various molecular mechanisms (103–105). Studies have revealed a close association between gastrointestinal dysfunction and the exacerbation of neurological disorders. For example, the most important non-neurological complications of Huntington's disease are associated with gastrointestinal defects. Two studies depicted that the patients with symptomatic Huntington's disease exhibited serum metabolomic shifts that suggested changes in gut microbial-derived metabolites (106, 107). Additionally, a study examined at how curcumin interacted with the gut microbiota of APP/PS1 double transgenic mice from two perspectives. Curcumin administration improved spatial learning and memory abilities in these mice while also lowered amyloid plaque burden in the hippocampus. Interestingly, curcumin administration significantly altered the relative abundances of bacterial taxa such as *Bacteroidaceae, Prevotellaceae, Lactobacillaceae*, and *Rikenellaceae* at the family level, and *Prevotella, Bacteroides*, and *Parabacteroides* at the genus level, several of which have been reported to be key bacterial species associated with Alzheimer's disease (AD) development. It is important to note that, the gut microbiota of AD mice produced a total of 8 curcumin metabolites, and many of these metabolites have been shown to have neuroprotective properties (108).

Xu et al. used next-generation sequencing technology to investigate the effects of curcumin on gut microbiota in a rat model of uric acid nephropathy (UAN). As a result, they found that in the interventional group, renal pathological lesions were reduced, and the serum uric acid and metabolic endotoxemia were decreased, which were tightly connected with a suppressed proliferation of opportunistic pathogens such as *Escherichia coli* and *Bacteroides*, and increased relative abundance of bacteria that produce short-chain fatty acids (SCFAs), such as *Lactobacillus* and *Ruminococcaceae* (109). In a clinical trial, curcumin supplementation for 6 months restored the α-diversity of gut microbiota to a normal value in the patients diagnosed with chronic renal disease (CKD). At the phylum level, curcumin supplementation significantly inhibited the growth of *Escherichia Shigella*, but enhanced that of *Lachnoclostridium*. Moreover, compared to the baseline levels, *Lactobacillaceae* spp. were found to be considerably higher at the family level in the last 3 months of supplementation. No adverse events were reported in the supplemented group, suggesting a high safety profile of curcumin phytosome after long-term dosing (110).

Colorectal cancer (CAC) progression is also influenced by gut bacterial profile. In an IL-10 knockout CAC mouse model, a curcumin-rich diet significantly improved survival, reduced colon weight/length ratio, and eradicated total tumor burden. Such beneficial effects were associated with an enhanced bacterial diversity, mitigated age-related decreases in alpha diversity, a raised *Lactobacillales* relative abundance, and a decreased *Coriobacterales* composition. Therefore, the anti-tumorigenesis properties of curcumin were linked to the maintenance of a more diversified colonic microbial ecology (111).

## Anthocyanins and gut microbiota

### Sources and chemical structures of anthocyanins

**Figure 5:**
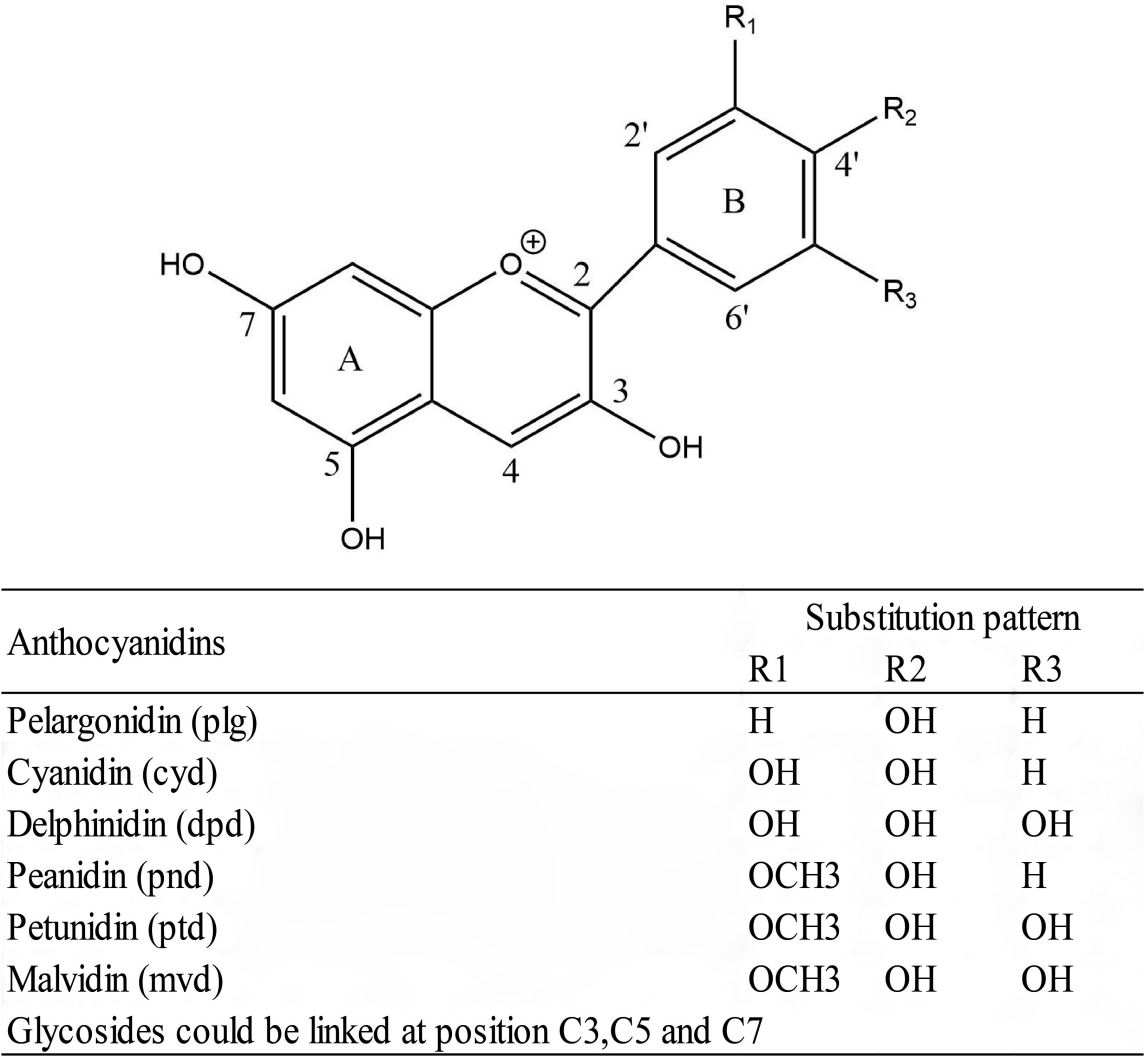
General structure of anthocyanidins and substitution pattern.

Anthocyanins are a class of flavonoids found in nature as pigments in a wide range of plant food. It is abundant in many berry varieties, including blackberries, blueberries, and cranberries. Their color changes depending on the pH of the food matrix and may appear purple, red, or blue. Anthocyanins contain a phenolic structure that contributes to their biological effects. Pelargonidin, cyanidin, delphinidin, peanidin, petunidin, and malvain are common anthocyanins that occur naturally in food categories (Figure 5) (112, 113). Anthocyanins have been linked to a variety of positive health outcomes, including improved blood vessel function, cancer prevention, and bone health. Since anthocyanins are naturally present in dietary sources, their use in the prevention and treatment of adverse health events is of interest, and anthocyanins may provide a safe, inexpensive, and low-risk approach to disease prevention (114–116).

### Bioavailability and metabolism of anthocyanins

The bioavailability of anthocyanins can be defined as the fraction of anthocyanins that are absorbed and utilized by the body. The bioavailability of natural anthocyanins has been estimated to be as low as 2% (117). During metabolism, only a small fraction of anthocyanins are absorbed by the gastrointestinal tract and then transferred to various bodily tissues such as the kidney and liver. Meanwhile, considerable amounts of anthocyanins bypass the small intestine and enter the colon, where they are hydrolyzed and fermented by the microbiota for further digestion (118, 119). The resulting colonic metabolites of anthocyanins are transported to the liver to be further metabolized and subsequently distributed to the circulating system to achieve various biological effects.

### Interactions between anthocyanins and gut microbiota

Anthocyanin-rich diets may impact the composition of the gut microbiota, which acts as a modulator for anthocyanin-related health benefits. The metabolites of major anthocyanins have been shown to improve overall gut integrity by decreasing inflammation and oxidative stress (120).

The interactions between anthocyanins and gut microbiota are important factor to be considered to understand the biological activities of anthocyanins for their corresponding health benefits. The interlinkage between anthocyanin biotransformation to potentially more bioactive, low molecular weight metabolites and anthocyanin-mediated modification of gut microbiota composition contributes to favorable health outcomes (121).

### Metabolism of anthocyanins by gut microbiota

With a small number of dietary anthocyanins are directly absorbed, the majority of the ingested compounds reach the colon. *Bifidobaterium* spp. and *Lactobacillus* spp. are two major families of gut bacteria that have glucosidase activities. They can metabolize phenolic substances during growth, providing energy to foster the growth of other gut bacteria. These bacterial groups have been linked to positive effects in the large intestine, such as the antibacterial action of pathogenic microorganisms through the generation of short-chain fatty acids and competition for growth substrate and adhesion sites (112).

*In vitro* studies conducted by Tian et al. showed that the bacterial metabolism of anthocyanins involves glycosidic linkage cleavage, anthocyanidin heterocycle breakdown, and degradation into phloroglucinol derivatives and benzoic acids (122). Anthocyanins and metabolites produced in the intestine simultaneously have the potential to selectively stimulate or hinder certain bacterial growth. Different metabolites may be formed *via* anthocyanin fermentation depending on the bacterial composition. The maximum conversions of anthocyanins by probiotic bacteria have been recorded, with *L. plantarum* and *S. thermophiles* degrading cyanidin-3-glucoside and cyanidin-3-rutinoside, respectively (123). Microbial fermentation of anthocyanins and the catabolism of those compounds at various stages along the process may produce a wide range of chemicals, resulting in a highly dynamic profile of some molecules.

### Modulation of gut microbiota by anthocyanins

The colonization of the gut microbiota can be altered by anthocyanin ingestion, influencing intestinal bacterial proliferation. *Bifidobacterium, Lactobacillus*, and *Akkermansia* are among these bacteria that can benefit the host, and they can catalyze anthocyanins alongside *Bacteroides* and *Eubacterium* (124). These bacteria have been linked to beneficial effects in the large intestine, including the antimicrobial effect of pathogenic bacteria by producing short chain fatty acids and competing for growth substrate and adhesion sites, but they also reduce potentially harmful bacteria, such as *Clostridium histolyticum*, which has been linked to tumor promotion and inflammatory bowel disease (125).

In the mice with colon cancer, oral administration of bilberry anthocyanin extract (BAE) enriched the diversity of bacterial populations in the digestive system and increased the abundance of *Clostridium* and *Lactobacillus johnsonii*. Meanwhile, the improvement of gut microbiota with daily BAE supplementation has been shown to reduce tumor growth and improve PD-L1 treatment efficacy (126). Faria et al. reported that *in vitro* incubation of malvidin-3-glucoside with fecal slurry increased the growth of *Bifidobaterium* spp. and *Lactobacillus* spp., but had no effect on the growth of *Bacteroides* spp. Surprisingly, adding malvidin-3-glucoside to other anthocyanins displayed a synergistic effect on bacterial growth. Gallic acid, an anthocyanin metabolite found in the gut, has been shown to reduce the abundance of potentially harmful bacteria including *Clostridium histolyticum* without affecting healthy bacteria (112).

### Anthocyanins, microbiota composition, and related health benefits

It has been shown that anthocyanins are able to prevent or delay the onset of certain diseases with their anti-inflammatory and antioxidative properties (127). Phenolic chemicals have the capacity of blocking pro-inflammatory mediators and thereby reducing inflammation. Anthocyanin metabolites have been shown to activate Nrf2, which activates the antioxidant enzymes and antioxidation-related pathways. They may also lower intestinal inflammation by modulating the MAKP and NF-B pathways mediated by TAK1 and SphK/S1P (113). Hidalgo et al. portrayed that a mixture of anthocyanins enhanced the growth of *Bifidobacterium* and *Lactobacillus-Enterococcus* in a batch culture fermentation system resembling the human distal large intestine, and interestingly, the microbial metabolite malvidin-3 -glucoside alone displayed the similar beneficial effects compared with its parent compounds (128).

In a study by Liu et al. oral administration of anthocyanins in the C57BL/6 J mice significantly reduced high fat diet-induced body weight gain by 20–27%, total adipose tissue weight by 18–25%, and plasma total cholesterol by 25%. Meanwhile, they observed a significantly reduced plasma lipopolysaccharide concentration, which was correlated with decreased relative abundances of *Rikenella* and *Rikenellaceae*. At the genus level, dietary supplementation of berry anthocyanin extract enhanced the grwoth of the groups of *Lachnoclostridium, Roseburia*, and *Clostridium innocuum*, resulting in increased fecal short-chain fatty acid (SCFA) release (129). Consistently, two studies found that anthocyanins might be able to alleviate high fat diet-induced dysbiosis by reducing *Luminococcus* (129) and *Muribaculaceae* (130), while enriching *Oscillobacter* (129).

Anthocyanins may prevent neurodegenerative illnesses by modulating the microbiome in the gut. Marques et al. explored into this association in rats fed a high fat diet, which is known to contribute to obesity-related neuroinflammatory and neurobehavioral abnormalities *via* altering the gut flora. Increased *Oscillibacter* were discovered in the gut of the animals treated with the anthocyanin-rich blackberry extract. They came to the conclusion that anthocyanin regulation in the gut is linked to anti-neuroinflammatory properties by lowering TCK-1 expression, and that anthocyanins might affect the central nervous system by altering tryptophan metabolism in the kynurenine pathway, thereby increasing the production of neuroprotective metabolites and reducing systemic inflammation (130).

Khan et al. recently shown that anthocyanins can lower the expression of proinflammatory cytokines, preventing against the pathogenesis of neuroinflammation and Alzheimer's disease (131, 132). Furthermore, anthocyanin has been shown to protect against Alzheimer's disease and synapsis-related functions in A1-42-injected mice (133). By blocking α'-amylase, anthocyanins may limit starch digestion. When this undigested starch enters the large intestine, it feeds probiotic bacteria like *Lactobacilli, Bifidobacteria*, and *Streptococci*, allowing them to continue to boost health (134).

## Quercetin and gut microbiota

### Sources and chemical structures of quercetins

**Figure 6:**
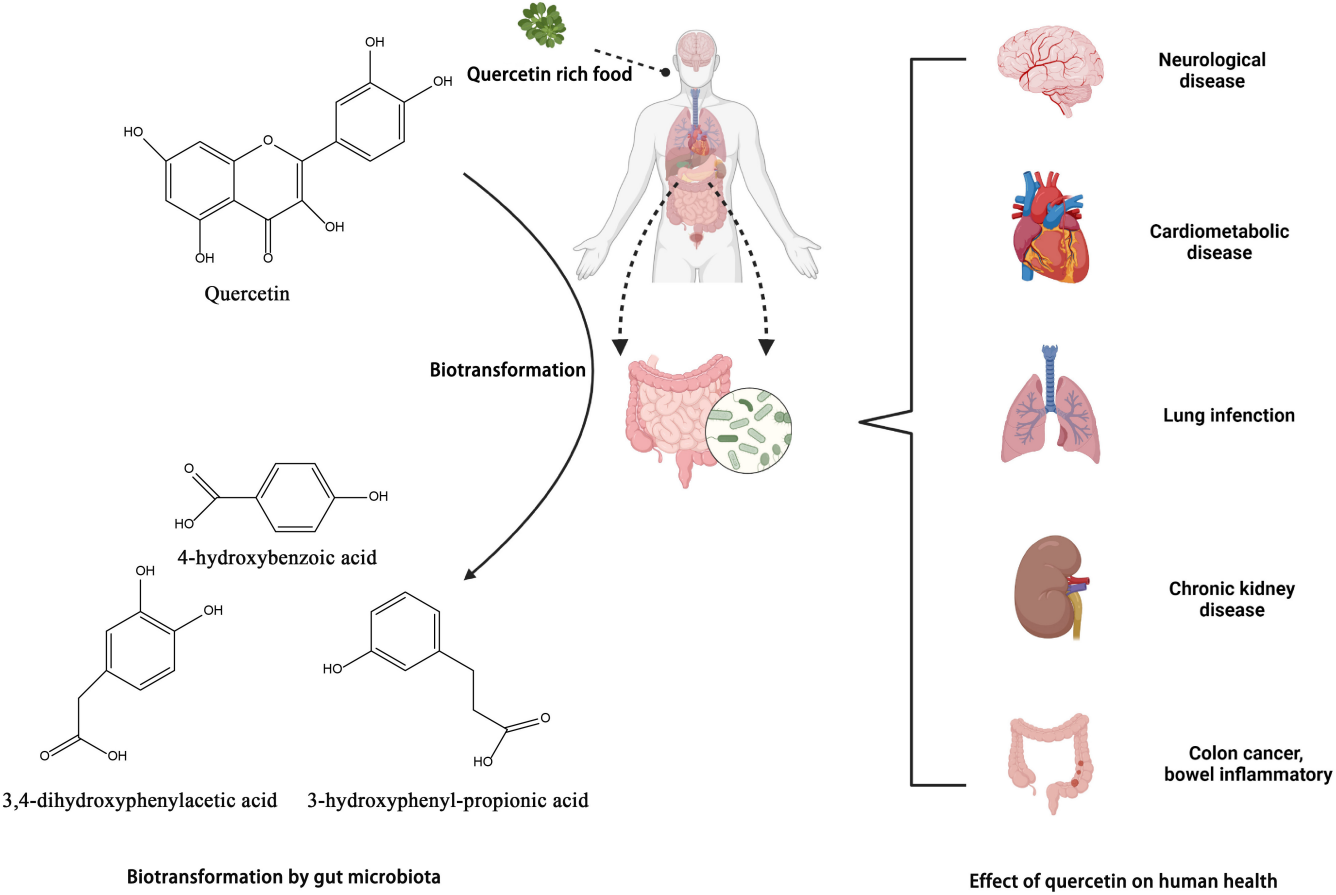
Biotransformation of quercetin into metabolites by gut microbiota and their benefits in gut. Created with BioRender.com.

Quercetin, a polyphenolic flavonoid, is abundantly present in onions, kale, apples, cherries, and red wine. Quercetin binds to sugar moieties like rhamnose or rutose in nature by attaching a sugar group as a substitute for one of the OH groups, forming quercetin glycosides and rutin (135). The sugar groups linked to quercetin can change its solubility, bioavailability, and bioactivities (136). Quercetin's antioxidative properties are essential in the prevention and treatment of illnesses. Different pharmacological benefits of quercetin in treating osteoporosis, blood pressure, cancer, and cardiovascular disease have been described in animal and human research (136, 137) (Figure 6).

### Bioavailability and metabolism of quercetins

Based on human studies, oral quercetin is primarily administered as a purified aglycone supplement. The typical daily consumption of quercetin in China, the United States, and Europe is 6–18 mg (54, 135). The bioavailability of quercetin after a single oral administration was found to be relatively low in human pharmacokinetic trials. In healthy people, the absorption rate of quercetin glucoside (the natural form of quercetin) varies between 3 and 17%/100 mg. Limited absorption, extensive metabolism, and/or quick excretion may all contribute to quercetin's low bioavailability (136).

Quercetin metabolism occurs primarily in the liver. After absorption, quercetin is transported to the liver, where it undergoes phase I and phase II metabolism, producing metabolites that circulate in the blood for distribution to body tissues (138). To fully understand the concentration of quercetin in plasma following repeated administration of quercetin-rich meals, Mullen et al. evaluated the accumulation of quercetin conjugates in human plasma after feeding the subjects with onions on a regular basis. The participants consumed about 100 mg of quercetin each meal from onion slices over three meals for a week. Results showed that fasting plasma levels of glucuronide and sulfate metabolites in participants increased from 0.04 to 0.63 μM. The primary plasma metabolites quercetin-30-sulfate and−3-glucuronide reached their peak levels after half an hour. Furthermore, after 4 h, the major urine metabolites quercetin-diglucuronide,−3′-glucuronide, isorhamnetin-3-glucuronide, and sulfate-glucuronide all peaked (139). Compounds that were not absorbed in the small intestine escaped to the large intestine, where colonic microbiota degraded the quercetin into phenolic acid compounds that were easily absorbed and delivered to the liver *via* the portal vein (140).

### Interactions between quercetins and gut microbiota

In the intestine, quercetin is metabolized by resident microbiota, and the resulting products may have different biological activities compared with the parent compound. Reciprocally, quercetin may alter the microbiota's composition, which is one of the proposed mechanism of actions for these chemicals. Through promoting beneficial flora and inhibiting potentially pathogenic flora, quercetin can therapeutically target gut microbiota and produce beneficial health consequences for the human host (141).

### Metabolism of quercetins by gut microbiota

In the intestine, quercetin is converted by the microbiota to 3,4-dihydroxyphenylacetic acid, also known as 3-(3-hydroxyphenyl) propionic acid, 3,4-dihydroxybenzoic acid, and 4-hydroxybenzoic acid. *Bacteroides fragilis, Clostridium perfringens, Streptococcus, Lactobacillus, Bifidobacterium*, and *Eubacterium cladobacterium* have been identified as the strains that convert quercetin to the compounds indicated above (54, 142). Jaganath et al. analyzed the microbial metabolites of quercetin 3-O-rutinand suggested that they might enhance the overall antioxidant capacity of the colon following ingestion of quercetin-rich food (143).

### Modulation of gut microbiota by quercetins

Quercetin has a significant impact on the gut environment, which in turn has an impact on the regulation of gut microbiota. Food-pathogenic bacteria such as *Staphylococcus aureus, Escherichia coli, Listeria monocytogenes*, and *Vibrio parahaemolyticus*, as well as clinically important hospital or community-associated pathogens, may be present in the human gut microbiota. With the prebiotic and antibacterial effects, quercetin may diminish these pathogenic microbiota (144–146).

Lan et al. discovered that quercetin treatment improved the diversity of gut microbiota, and the major changes in the gut microbiota (*Clostridium, Bacteroides*, and *Bacilli*) were observed at the class level. After quercetin treatment, there was an increase in *Lactobacillus* and a decrease in *Ruminococcus* (144). Meanwhile, Shi et al. discovered that quercetin supplementation improved the diversity of the gut bacterial community in antibiotic-treated mice. This was accompanied with an increase intestinal barrier function, as the researchers observed a decreased serum D-lactic acid concentration and serum diamine oxidase activity. Intestinal villi length and mucosal thickness were both increased considerably after quercetin supplementation. In addition, quercetin promoted rebuilding of mice's gut microbiota following antibiotic therapy and might be used as a prebiotic in the fight against gut dysbiosis (147).

### Quercetin, microbiome composition, and related health benefits

By using a mouse model of atherosclerosis and feeding the mice with quercetin for 12 weeks, researchers found that quercetin could prevent damaged arteries caused by a high fat diet, which was associated with significantly altered *Bacteroidetes, Firmicutes*, and *Proteobacteria* as well as increased *Phascolarctobacterium* and *Anaerovibrio* (148). Nie et al. found that in mice fed with a high fat diet for 12 weeks, oral quercetin supplementation significantly enhanced the immune/inflammatory responses and alleviated oxidative stress. Microbial analysis performed at the phylum level reported that quercetin treatment decreased the abundance of *Verrocomicrobia*, while increased the diversity of the microbiota and the abundance of *Actinobacteria, Cyanobacteria*, and *Firmicutes*. In addition, quercetin decreased gut cholesterol, lysophosphatidic acid, and atherogenic lysophosphatidylcholine levels, while increased faeprostanol levels (149).

Additionally, Lan et al. studied the effect of quercetin in alleviating osteoarthritis in a rats, and found that the quercetin supplementation reversed osteoarthritis-associated dysbiosis and changed the microbial metabolites. These studies suggested that quercetin might be used as a potential therapeutic approach in alleviating various diseases by modulating gut microbiota (144).

## Chlorogenic acid and gut microbiota

### Sources and chemical structures of chlorogenic acid

**Figure 7:**
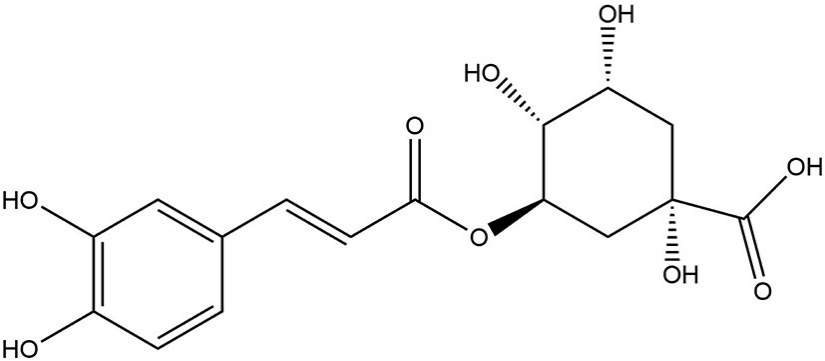
Chemical structure of chlorogenic acid.

Chlorogenic acid (CGA), one of the most common polyphenols in the human diet, is present in a variety of fruits, vegetables, and herbs, including apples, coffee beans, tea, and wormwood plants, and has a number of health-promoting qualities (150, 151). Chlorogenic acid belongs to the hydroxycinnamic acid family of phenolic compounds. With a caffeic acid (CA) and a quinic acid (QA) moiety, it is also known as 5-O-caffeoylquinic acid (5-CQA) (Figure 7) (152). Chlorogenic acid has shown several beneficial effects with its anti-oxidative, anti-inflammatory, anti-cancer and anti-neurodegenerative activities (150).

### Bioavailability and metabolism of chlorogenic acid

The hydrophilic characteristic of chlorogenic acid prevents it from passing through lipophilic membrane barriers after oral administration, resulting in limited absorption and bioavailability (153). It has been well-documented that chlorogenic acid is accessible and processed differently in the gastrointestinal tract, liver, and kidney in humans. In general, the metabolic pathways of chlorogenic acid are as follows: (I) in humans, around one-third of the chlorogenic acid in food is absorbed intact and enters the bloodstream without being hydrolyzed in the stomach and/or upper gastrointestinal tract; (II) a small amount of chlorogenic acid (about 7%) is absorbed throughout the small intestine, including hydrolysis to caffeic and quinic acid; (III) colonic microbiota-mediated metabolism of chlorogenic acid and absorption of metabolites; and (IV) intact chlorogenic acid and its metabolites are absorbed and/or metabolized in the liver (7, 154). Experiments in rat models have shown that chlorogenic acid is rarely hydrolyzed in the stomach and has no significant bioavailability before reaching the colon (155). Most chlorogenic acids are transferred to the large intestine and become small molecules with higher biological activities through the decomposition and metabolism by intestinal flora.

### Interactions between chlorogenic acid and gut microbiota

Chlorogenic acid is formed from the esterification of caffeic acid and quinic acid and is often referred to as a “non-polysaccharide” based prebiotic. Only a small fraction of ingested chlorogenic acid is absorbed by the small intestine, while the majority is metabolized in the large intestine where it is extensively degraded by the gut microbiota. The gut microbiota can break down chlorogenic acid into a series of low-molecular-weight aromatic acid metabolites, including metacoumaric acid and derivatives of phenylpropionic acid and benzoic acid, which may be the main molecules responsible for its biological activity. For example, benzoic acid may support gut health by modulating gut microboita (156–158).

### Metabolism of chlorogenic acid by gut microbiota

The colon's resident microflora could quickly hydrolyze chlorogenic acid, and the resultant products could be further metabolized by the hosts' enzymes to produce additional metabolites that are then released to the circulating systems. When espresso coffee was cultivated with human stool samples for 6 h, it was discovered that the colonic microbiota swiftly decomposed all of the coffee chlorogenic acids, resulting in a total of 11 catabolites (155).

Tomas-Barberan et al. conducted resting cell studies on nine different colonic populations to investigate the biotransformation of caffeoylquinic acid by diverse human colonic microbiota. Before hydrolyzing their ester linkages, different bacterial communities can hydrogenate, dehydroxylate, or eliminate the quinic acid moiety. All conversion pathways focus on 3-(3-hydroxyphenyl)-propionic acid (HPPA), which is the final metabolite in most samples. The transformation rate of caffeoylquinic acid was increased under the action of *Bifidobacterium animalis subsp*. *lactis* (159).

It has also been reported that nearly 30% of ingested 5-CQA is hydrolyzed to caffeic acid and quinic acid in the rat cecum (7). These findings are in line with the *in vitro* evidence that human fecal bacteria at least partially hydrolyze 5-CQA.

### Modulation of gut microbiota by chlorogenic acid

Chlorogenic acid may regulate the relative abundances of some essential microbial species (e.g., *Burkholderiales, Desulfovibrio, Klebsiella, Desulfovibrionales*, and *Bifidobacterium*, among others), which consequently benefit the host (155).

An *in vitro* study showed that chlorogenic acid significantly increased the abundance of beneficial bacteria such as the *Bifidobacterium* spp and the *Clostridium coccoides-Eubacterium rectale* group (153). Chen et al. discovered that chlorogenic acid treatment greatly enhanced the abundance of the phylum *Firmicutes*, adding to our knowledge of the favorable effects of dietary chlorogenic acid on improving nutrient bioavailability (158). Meanwhile, dietary chlorogenic acid supplementation reduced *Bacteroides* abundance in ileal samples from weaned pigs, while *Bacteroides* are gram-negative obligate anaerobic bacteria that have deleterious effects on the gut (156). Another study conducted by Ludwig et al. showed that the abundance of bacteria from the phylum *Proteobacteria* in the cecal samples of the chlorogenic acid group was significantly lowered. This is important because most bacteria in the *Proteobacteria* phylum have been shown to cause long-term intestinal inflammation and injury in both neonates and adults (160).

### Chlorogenic acid, microbiome composition, and related health benefits

Chlorogenic acid, as well as its metabolites caffeic acid and ferulic acid, are potent antioxidants. One study by Ding et al. demonstrated that chlorogenic acid could efficiently ameliorate cadmium-induced kidney and liver damage. Oral chlorogenic acid supplementation significantly enhanced the abundance of *Rikenella* and other anti-inflammatory bacteria, lowering cadmium poisoning symptoms (161). Ye et al. investigated the role of gut microbiota in the protection of obesity and metabolic endotoxemia by supplementing C57BL/6 mice with chlorogenic acid. As a result, chlorogenic acid altered the gut microbiota's composition by increasing the abundance of SCFA-producing bacteria (e.g., *Dubosiella, Romboutsia, Mucispirillum*, and *Faecalibaculum*) and *Akkermansia*, which was correlated with enhanced intestinal barrier. Furthermore, animals with a microbiota that was modified by chlorogenic acid had reduced body weight and fat content, as well as developed less metabolic endotoxemia (162).

Another study by Wang et al. found that administering chlorogenic acid for 6 weeks reduced body weight, improved plasma lipids linked with high fat diet-induced obesity, and modulated lipogenesis and adipogenesis gene expressions in the epididymal white adipose tissue (163). Furthermore, chlorogenic acid treatment significantly decreased *Desulfovibrio, Erysipelas, Lachnospira*, and *Ruminococcus*, as well as increased *Bacteroidetes* and *Lactobacilli*, which was associted with a decreased obesity severity. Shi et al. aimed to investigate the effect of chlorogenic acid on high fat diet-induced mouse model of non-alcoholic fatty liver disease (164), and reported that chlorogenic acid could reduce high fat diet-induced hepatic steatosis and inflammation, lower serum transaminases, and improve insulin sensitivity. Simultaneously, chlorogenic acid increased the *Bifidobacteria* content while decreased the *Escherichia coli* content in feces. These findings suggest that chlorogenic acid, through its capacity for modulating gut microbiota, may protect against high fat diet-induced hepatic steatosis and inflammation.

Bhandarkar et al. hypothesized that chlorogenic acid could improve cardiovascular, hepatic, and metabolic responses in a high carbohydrate, high fat diet-induced rat model of metabolic syndrome. Energy intake and food absorption efficiency were reduced in rats with chlorogenic acid supplementation, resulting in a reduction in visceral fat. With these, chlorogenic acid may improve the overall metabolism by regulating the diversity and profile of gut microbiota. Thus, long-term dietary chlorogenic acid reduces diet-induced inflammation as well as cardiovascular, hepatic, and metabolic changes, indicating the potential for further clinical studies of chlorogenic acid (165). However, there is a lack of well-designed clinical trials that validated the efficacy of chloric acid in improving metabolic health.

## Current research and limitation

**Figure 8:**
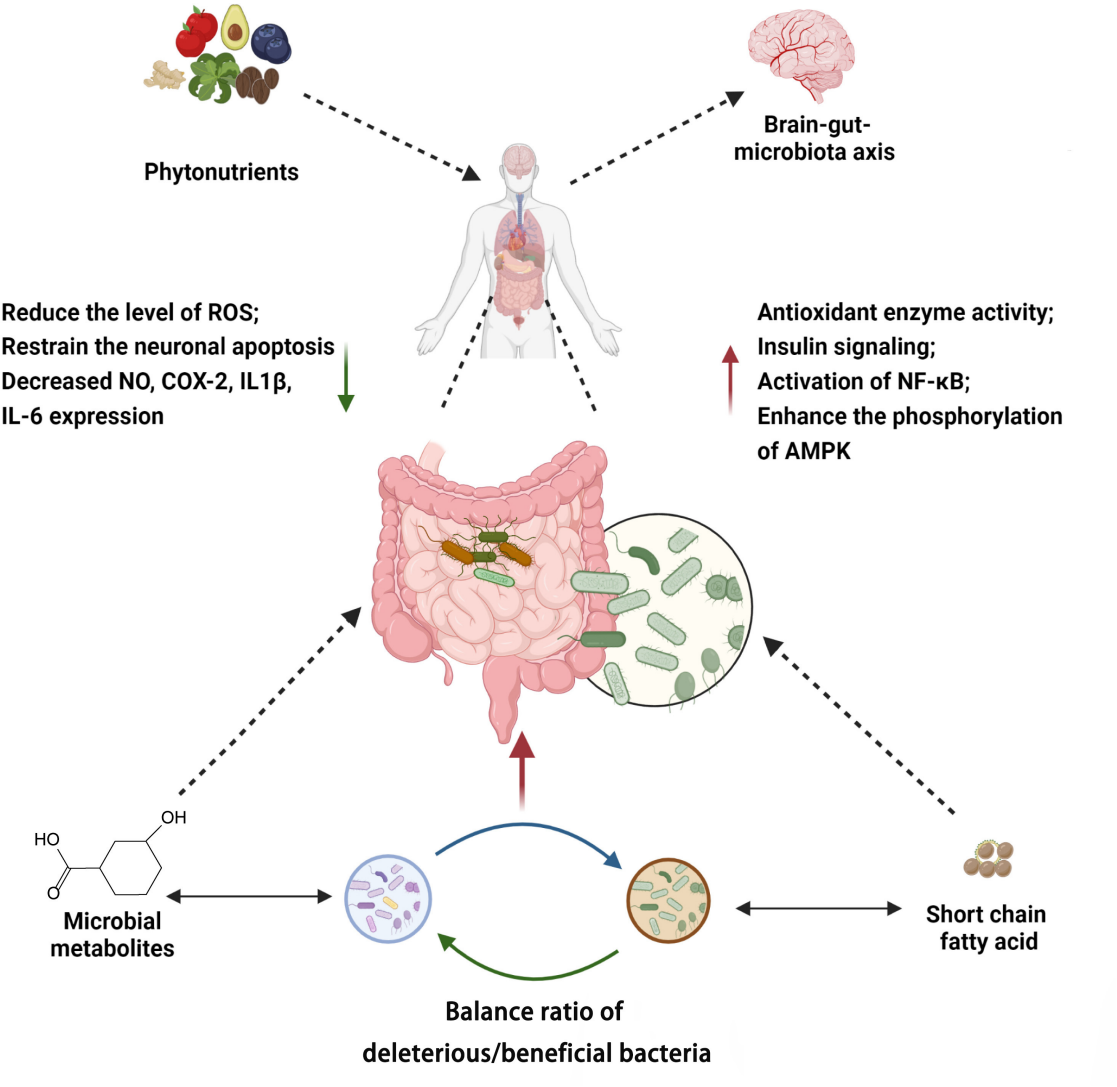
Summary of mechanisms associated with phytochemicals modulating gut microbiota. Created with BioRender.com.

Phytonutrients modulate the gut microbiota through multiple mechanisms. These pathways may act independently or in crosstalk (Figure 8, Supplementary Table 1). Taken together, the mechanisms by which phytonutrients interact with gut microbiota and in turn affect human health include (I) direct or indirect regulation on the composition of the gut microbiota, which has an impact on the brain-gut-microbiota axis and other processes, (II) metabolism by gut microbiota, which enhances bioavailability and bioactivity of the phytonutrients, and (III) synergistic activities of different types of phytonutrients.

By reviewing the roles and mechanisms of the representative phytonutrients above, we conclude that the interactions between phytonutrients and gut microbiota may confer multiple benefits to human health (Table 1). However, various factors continue to obstruct phytonutrient function, including phytonutrient bioavailability, the composition of the gut microbiota, and the processes through which the gut microbiota influences the human body.

**Table 1:** Effect of phytonutrients on gut microbiota modulation and their major effects on human health.

**Phytonutrient**	**Source**	**Type of study**	**Change to microbiome**	**Outcome of benefit**	**References**
Catechins	Tea, cacaos, apples, berries, grapes	Fermentation *in vitro, in vivo* animal study	(+) *Bifidobacteria* (–) *Bacteroides* *Prevotella*, *Clostridium* *histolyticum* *Eubacterium-Clostridium*	Alleviate inflammation, resist microbial invasion, regulate immunity, protect the nervous system, protect circulatory system and cardiac tissues	(70, 166)
EGCG	Green tea	*In vitro* assay in bacterial medium, *in vivo* animal and human study	(+) *Bacteroides, Christensenellaceae, Bifidobacterium* (–) *Firmicutes, Bacteroidetes*	Prevent GMD, suppress obesity, modulates gut microbiota, alleviate low-grade inflammation	(167–169)
Polyphenol	Green tea	*In vivo* animal study and human study	(+) *Firmicutes* and *Bacteroidetes*	Reduce weight, promote energy conversion, reduce the levels of glucose, triglycerides and total cholesterol	(170, 171)
Curcumin	Turmeric	Pilot study in humans	(–) *Clostridium* *Bacteroides* *Citrobacter* *Cronobacter* *Enterobacter* *Enterococcus* *Klebsiella*	Resistance to inflammation, neurotrophic effects, restore normal gut microbial diversity	(93)
Anthocyanins	Cranberry, grape	*In vitro* (batchculture fermentation)	(–) *Bacteroides, Prevotella, Blautia, Lactobacillus*, *Bifidobacterium*, *Enterobacteriaceae*, *Ruminococcus*	Control blood sugar, reduce insulin resistance, reduce inflammation	(172)
Quercetin	Green tea, lettuce, cranberry, apple, onion,	*In vivo* animal and human study	(–) *Firmicutes, Erysipelotrichia* and *Bacillus genus, Bacillus, Eubacterium cylindroides*, *Erysipelotrichaceae*	Reduce inflammation, reduce insulin resistance	(173, 174)
Quercetin	Onions, tea, lettuce, broccoli, apples	*In vitro* (human feces)	(–) *Escherichia coli, Streptococcus, lutetiensis*, *Enterococcus gilvus, Clostridium perfringens, Bacteroides fragilis, Lactobacillus acidophilus*	Converted to beneficial metabolites by *C. perfringens* and *B. fragilis*	(142)
Chlorogenic acid	Ertain fruits, vegetables	*In vivo* animal model	(+) *Bifidobacterium, Dubosiella, Romboutsia, Mucispirillum, Faecalibaculum, Akkermansia* (–) *Escherichia coli*	Reduce oxidative stress, reduce inflammation, reduce acute lung injury, protect cardiovascular	(162, 164)
Coffee and Caffeic acid	Green and roasted coffee beans, or red wine	*In vivo* animal and human study	(+) *Bifidobacterium spp*.	Prevention of colon cancer metastasis, inhibit tumor cell transformation	(175, 176)

The physiochemical parameters that affect bioavailability of phytonutrients include the chemical class, polarity, molecular weight, structure of the phytonutrients, the activity of gastrointestinal enzyme, and the enterocyte absorption (177). Administering phytonutrients by modifying them into appropriate dosage forms would be a strategy to improve the efficiency of phytonutrient metabolism and absorption. Chen et al. proposed the use of novel nano-formulation techniques to target delivery of these phytonutrients. The cumulative transport and bioavailability of nano-EGCG was about twice of free EGCG, and the dose advantage of nano-EGCG to induce apoptosis in prostate cancer cells was more than ten times (178).

In addition, the bioavailability of most phytonutrients depends on the microbes in the gut. By regulating the abundance and quantity of gut microbiota, the bioavailability of phytonutrients can be effectively improved. For example, when mice were fed with a diet rich in pectin-rutin, bacterial abundances in *Bacteroidetes, Clostridium, Eubacteria, Enterobacteriaceae, Lactobacillus*, and *Streptococcus* were significantly increased, and plasma quercetin level was also increased (179). Meanwhile, dietary phytonutrients were metabolized by gut microbiota to form beneficial short-chain fatty acids, which might differentially affect phytochemical bioavailability by modulating gut microbiota.

Furthermore, while earlier research has explored the impact of phytonutrients on gut microbial composition, little is known about the synergic effects of intestinal epithelial integrity and microbiota in regulating the bioavailability and bioactivity of phytonutrients. Additionally, most research focused on fecal samples, which may not accurately reflect the entire small intestinal or cecal microbiome. In order to better depict the dynamic changes of the gut microbiota and the intestine, an appropriate animal model can be established for research. For instance, Sun et al. investigated the interaction of curcumin with gut microbiota in APP/PS1 double transgenic mice for the treatment of Alzheimer's disease. The findings suggested that curcumin could affect the diversity of the gut microbiota, and a range of metabolites were bio-transformed by the gut microbiota, which would provide insights into the treatment of Alzheimer's disease (108). By addressing these questions, we will have a clear understanding of the relationship between phytonutrients and gut microbiota, as well as the mechanism by which gut microbiota acts on human body, which will guide the use of phytonutrients in the future.

## Conclusion and prospects

Phytonutrients, as a class of nutritional supplements that can be obtained from the daily diet, enter the body through oral absorption, produce biologically active substances during metabolism in the body, and regulate the abundance and composition of intestinal flora. In this review, we focus on phytonutrients, including catechins, curcumin, anthocyanins, quercetin, and chlorogenic acid, to discuss their sources, bioavailability, interaction with gut microbiota, and their impacts on human health. Although the recent two decades witnessed an increasing interest in the studies of phytonutrients and microbiota, the research on the interaction between phytonutrients and gut microbiota is still in its infancy. In the foreseeable future, phytonutrients may be applied to pregnant women to maintain gut health, promote neurodevelopment, and benefit overall wellness of the infant. At the same time, the application of phytonutrients in the formation of intestinal flora in children can help mitigate adverse conditions such as inflammation in body, thereby enabling health promotion at the adolescent stage. In addition, phytonutrients may be used to improve sub-health status in adults, and daily supplementation of corresponding phytonutrients may have preventive and therapeutic effects. Although phytonutrients have shown various beneficial effects, more studies are warranted to explore how gut microbiota may enhance these bioactivities, as well as the interaction between phytonutrients and gut microbiota. It would also be intriguing to explore unbeknown potential beneficial effects of phytonutrients and their microbial metabolites in the prevention and/or mitigation of diseases.

## Author contributions

YY and JD designed the manuscript. FWa, JZ, and YL performed the literature review. FWu and JK drafted the manuscript. JK and JC revised the manuscript. All authors contributed to the article and approved the submitted version.

## Conflict of interest

Authors JK, FWa, JZ, and JD are employees of Nutrilite Health Institute, a division of Amway. Authors FWu and YL are employed by Sequanta Technologies Co., Ltd. The remaining authors declare that the research was conducted in the absence of any commercial or financial relationships that could be construed as a potential conflict of interest.

## Publisher's note

All claims expressed in this article are solely those of the authors and do not necessarily represent those of their affiliated organizations, or those of the publisher, the editors and the reviewers. Any product that may be evaluated in this article, or claim that may be made by its manufacturer, is not guaranteed or endorsed by the publisher.
