# Supplementary material for: Phytonutrients: Sources, bioavailability, interaction with gut microbiota, and their impacts on human health
Source: Front Nutr. 2022 Aug 16;9:960309. doi: 10.3389/fnut.2022.960309 (PMC9424995; doi:10.3389/fnut.2022.960309)
Supplement: Supplementary file 1 [file Table_1.DOCX]

**Supplementary Table 1.** Summary of the modulatory mechanism of phytonutrients on gut microbiota

| **Phytonutrient** | **Modulatory effect** | **Mechanism of action** | **Ref** |
| --- | --- | --- | --- |
| Polyphenols | Reduction of medical chronic conditions | Enhance phosphorylation of AMPK by affecting the symbiotic microbiota | (1) |
| Green tea catechins | Reduce atherosclerotic disease risk factors, and pro-inflammation | Reduce the levels of reactive oxygen species (ROS) produced by hemodialysis | (2) |
| EGCG | Treatment of diabetes, neurological diseases, cognitive dysfunction | Enhance the insulin signaling and dispel oxidative stress;  restrain the neuronal apoptosis, oxidative stress injury and brain damage marker levels | (3, 4) |
| Flavanols | Resistance to oxidative stress-induced nerves damage | Activate intracellular antioxidant enzyme activity by blocking radical chain reaction, and chelate iron or copper ions to carry out free radical scavenging | (5) |
| Curcumin | A relative increase in *Lactobacillus* abundance and a decrease in *Corybacteriaceae* | Suppress mucosal mRNA expression of inflammatory mediators and the activation of NF-κB in colonic epithelial cells; inhibit the proliferation and induce apoptosis through the COX-2 and non-COX-2 pathways | (6, 7) |
| Quercetin | Increase Nrf2-mediated GCLC/GCLM expression, increase GSH content in cells; inhibit lead-induced endoplasmic reticulum stress | Induce Nrf2/GCL/GSH antioxidant signal transduction pathways; reduce oxidative stress in liver, inhibit JNK phosphorylation, and increase PI3K and Akt levels | (8, 9) |
| Anthocyanins | Attenuate inflammatory responses | Decrease NO, COX-2, IL1β, IL-6 expression; suppress NF-kB, AP-1, MAPK/JNK inflammatory pathways | (10) |
| Chlorogenic acid | Prevention and treatment of metabolic syndrome and associated disorders | Scavenge ROS, suppress the expression of inflammation | (11, 12) |

References

1. Xing L, Zhang H, Qi R, Tsao R, Mine Y. Recent Advances in the Understanding of the Health Benefits and Molecular Mechanisms Associated with Green Tea Polyphenols. J Agric Food Chem. 2019 Jan 30;67(4):1029-1043. Epub 20190117. doi:10.1021/acs.jafc.8b06146. Cited in: Pubmed; PMID 30653316.

2. Hsu SP, Wu MS, Yang CC, Huang KC, Liou SY, Hsu SM, Chien CT. Chronic green tea extract supplementation reduces hemodialysis-enhanced production of hydrogen peroxide and hypochlorous acid, atherosclerotic factors, and proinflammatory cytokines. Am J Clin Nutr. 2007 Nov;86(5):1539-47. doi:10.1093/ajcn/86.5.1539. Cited in: Pubmed; PMID 17991670.

3. Khan N, Mukhtar H. Tea Polyphenols in Promotion of Human Health. Nutrients. 2018 Dec 25;11(1). Epub 20181225. doi:10.3390/nu11010039. Cited in: Pubmed; PMID 30585192.

4. Pervin M, Unno K, Takagaki A, Isemura M, Nakamura Y. Function of Green Tea Catechins in the Brain: Epigallocatechin Gallate and its Metabolites. Int J Mol Sci. 2019 Jul 25;20(15). Epub 20190725. doi:10.3390/ijms20153630. Cited in: Pubmed; PMID 31349535.

5. Grau-Bove C, Gonzalez-Quilen C, Terra X, Blay MT, Beltran-Debon R, Jorba-Martin R, Espina B, Pinent M, Ardevol A. Effects of Flavanols on Enteroendocrine Secretion. Biomolecules. 2020 Jun 1;10(6). Epub 20200601. doi:10.3390/biom10060844. Cited in: Pubmed; PMID 32492958.

6. Ohno M, Nishida A, Sugitani Y, Nishino K, Inatomi O, Sugimoto M, Kawahara M, Andoh A. Nanoparticle curcumin ameliorates experimental colitis via modulation of gut microbiota and induction of regulatory T cells. PLoS ONE. 2017;12(10):e0185999. Epub 2017/10/07. doi:10.1371/journal.pone.0185999. Cited in: Pubmed; PMID 28985227.

7. McFadden RM, Larmonier CB, Shehab KW, Midura-Kiela M, Ramalingam R, Harrison CA, Besselsen DG, Chase JH, Caporaso JG, Jobin C, Ghishan FK, Kiela PR. The Role of Curcumin in Modulating Colonic Microbiota During Colitis and Colon Cancer Prevention. Inflamm Bowel Dis. 2015 Nov;21(11):2483-94. Epub 2015/07/29. doi:10.1097/MIB.0000000000000522. Cited in: Pubmed; PMID 26218141.

8. Jin Y, Huang ZL, Li L, Yang Y, Wang CH, Wang ZT, Ji LL. Quercetin attenuates toosendanin-induced hepatotoxicity through inducing the Nrf2/GCL/GSH antioxidant signaling pathway. Acta Pharmacol Sin. 2019 Jan;40(1):75-85. Epub 20180619. doi:10.1038/s41401-018-0024-8. Cited in: Pubmed; PMID 29921882.

9. Liu CM, Zheng GH, Ming QL, Sun JM, Cheng C. Protective effect of quercetin on lead-induced oxidative stress and endoplasmic reticulum stress in rat liver via the IRE1/JNK and PI3K/Akt pathway. Free Radic Res. 2013 Mar;47(3):192-201. Epub 20130115. doi:10.3109/10715762.2012.760198. Cited in: Pubmed; PMID 23249147.

10. Li L, Wang L, Wu Z, Yao L, Wu Y, Huang L, Liu K, Zhou X, Gou D. Anthocyanin-rich fractions from red raspberries attenuate inflammation in both RAW264.7 macrophages and a mouse model of colitis. Sci Rep. 2014 Aug 29;4:6234. Epub 20140829. doi:10.1038/srep06234. Cited in: Pubmed; PMID 25167935.

11. Santana-Galvez J, Cisneros-Zevallos L, Jacobo-Velazquez DA. Chlorogenic Acid: Recent Advances on Its Dual Role as a Food Additive and a Nutraceutical against Metabolic Syndrome. Molecules. 2017 Feb 26;22(3). Epub 20170226. doi:10.3390/molecules22030358. Cited in: Pubmed; PMID 28245635.

12. Naveed M, Hejazi V, Abbas M, Kamboh AA, Khan GJ, Shumzaid M, Ahmad F, Babazadeh D, FangFang X, Modarresi-Ghazani F, WenHua L, XiaoHui Z. Chlorogenic acid (CGA): A pharmacological review and call for further research. Biomed Pharmacother. 2018 Jan;97:67-74. Epub 2017/10/29. doi:10.1016/j.biopha.2017.10.064. Cited in: Pubmed; PMID 29080460.
